# Supplementary material for: A consensus-based ensemble approach to improve transcriptome assembly
Source: BMC Bioinformatics. 2021 Oct 21;22:513. doi: 10.1186/s12859-021-04434-8 (PMC8532302; doi:10.1186/s12859-021-04434-8)
Supplement: Supplementary file 2 — Additional file 2. Supplementary Tables (S1–S13). [file 12859_2021_4434_MOESM2_ESM.pdf]

Table S1. Distribution of the number of alternative transcripts in the benchmark datasets.[a]

# of alternative transcripts per gene[b]	# of genes		
	No0-NoAlt	Col0-Alt	Human HG38
1[c]	18,947	9,109	8481
2	0	1,915	2393
3	0	514	795
4	0	168	288
5	0	41	77
6	0	17	30
7	0	3	17
8	0	2	6
9	0	0	2
10	0	1	1
11	0	0	3
12	0	0	0
13	0	0	2
14	0	0	0
15	0	0	3
Total # of transcripts	18,947	16,071	18,348
Total gene length (bp)	30,051,975	24,982,520	28,030,410
Total # of reads[d]	497,448,498	496,589,302	494,422,694
Average # of reads per transcripts (minimum ~ maximum)	26,255 (38~8,811,100)	26,550 (2~5,746,600)	26,940 (2~28,287,676)

[a]For each dataset, only the genes RNAseq reads covered the entire transcribed region are included.
[b]Only transcripts coding unique protein sequences are counted as alternative forms.
[c]These genes have no alternative splice forms.
[d]Total number of simulated RNAseq reads produced from each benchmark transcriptome.

Table S2. Experimental design used for assessing the transcriptome assembly performance.

Test	Assembly type	RNAseq dataset	Reference genome	Reference type	Alternative splicing
1	*De novo*	No0-NoAlt	-	No	No
2	*De novo*	Col0-Alt	-	No	Yes
3	*De novo*	Human HG38	-	No	Yes
4	Genome-guided	No0-NoAlt	No-0	Same	No
5	Genome-guided	No0-NoAlt	Col-0	Different	No
6	Genome-guided	Col0-Alt	Col-0	Same	Yes
7	Genome-guided	Col0-Alt	No-0	Different	Yes
8	Genome-guided	Human HG38	HG38	Same	Yes
9	Genome-guided	Human HG38	HX1	Different	Yes

Table S3. Performance analysis of *de novo* assemblers.[a]

Assembler	Actual[b]	Total[c]	Unique[d]	*TP*	*FP*	*FN*	Precision	Recall	*F*
Test 1: No0-NoAlt									
IDBA-Tran	18,875	22,813	22,813 (120.86)	8,344	14,468	10,531	0.3658	0.4421	0.4003
rnaSPAdes	18,875	30,478	27,713 (146.83)	10,034	17,679	8,841	0.3621	0.5316	0.4307
SOAPdenovo-Trans	18,875	30,010	29,896 (158.28)	11,118	18,758	7,757	0.3721	0.5890	0.4561
Trinity	18,875	23,644	23,519 (124.60)	12,057	11,462	6,818	**0.5126**	**0.6388**	**0.5688**
Test 2: Col0-Alt									
IDBA-Tran	15,508	20,522	20,447 (131.85)	6,021	14,426	9,487	0.2945	0.3883	0.3349
rnaSPAdes	15,508	48,241	31,494 (203.08)	7,556	23,938	7,952	0.2399	0.4872	0.3215
SOAPdenovo-Trans	15,508	23,013	21,371 (137.81)	7,281	14,090	8,227	0.3407	0.4695	0.3949
Trinity	15,508	20,542	19,409 (125.15)	9,252	10,157	6,256	**0.4767**	**0.5966**	**0.5299**
Test 3: Human HG38									
IDBA-Tran	17,669	20,955	20,954 (118.59)	6,154	14,800	11,515	0.2937	0.3483	0.3187
rnaSPAdes	17,669	24,400	21,244 (120.23)	7,637	13,607	10,032	0.3595	0.4322	0.3925
SOAPdenovo-Trans	17,669	22,499	22,005 (124.55)	5,933	16,072	11,736	0.2697	0.3358	0.2991
Trinity	17,669	22,051	21,278 (120.43)	8,764	12,514	8,905	**0.4119**	**0.4960**	**0.4500**

[a]All assemblers were run using the default settings. The best performance score for each dataset is shown in red boldface.
[b]Total number of transcripts in the benchmark transcriptome.
[c]Number of all contigs produced by the assembler.
[d]Number of unique contigs produced by the assembler. Proportion (%) of the number of transcripts in the benchmark transcriptome is shown in parentheses.

Table S4. Comparison of the isoform assembly performance using the simulated Col0-Alt dataset.

Category[a]	All[b]	IDBA-Tran	SOAPdenovo-Trans	rnaSPAdes	Trinity	Bayesembler	Cufflinks	Scallop	StringTie2
1: No alternative splicing, assembled	6,638	5,011	5,043	5,218	5,504	4,611	5,681	6,176	6,022
2: No alternative splicing, not assembled	2,471	4,098	4,066	3,891	3,605	4,498	3,428	2,933	3,087
3: Alternative splicing, none assembled	370	753	1,513	1,566	1,061	520	1,085	770	877
4: Alternative splicing, one assembled	146	1,780	789	718	360	381	722	257	329
5: Alternative splicing, more than one assembled	2,145	128	359	377	1,240	1,760	854	1,634	1,455
6: Alternative splicing, all assembled	1,965	93	233	231	880	1,460	605	1,320	1,113
7: At least 3 isoforms, all assembled	495	0	7	9	104	293	47	201	134

[a]Categories 1 and 2 include genes without alternative transcripts that were assembled (Category 1) and not assembled (Category 2) by each method. Categories 3-7 include those with alternative transcripts where no transcript was assembled (Category 3), only one transcript was assembled (Category 4), more than one transcript was assembled (Category 5), all transcripts were assembled (Category 6), and at least three transcripts were assembled (Category 7) by each method.

[b]All dataset includes merged assemblies including both genome-guided and *de novo*, across all kmers. See also Fig. S1 in Additional file 3.

Table S5. Performance analysis of *de novo* assemblers pooled across multiple kmer lengths.[a]

Assembler	Actual[b]	Unique[c]	*TP*	*FP*	*FN*	Precision	Recall	*F*
Test 1: No0-NoAlt								
IDBA-Tran	18,875	106,631	13,799	92,832	5,076	0.1294	0.7311	0.2199
rnaSPAdes	18,875	258,548	14,172	244,376	4,703	0.0548	0.7508	0.1022
SOAPdenovo-Trans	18,875	209,406	13,615	195,791	5,260	0.0650	0.7213	0.1193
Trinity	18,875	84,687	12,783	63,360	6,092	**0.1679**	0.6772	**0.2691**
Merged[3]	18,875	564,629	14,770	549,859	4,105	0.0260	**0.7825**	0.0506
Test 2: Col0-Alt								
IDBA-Tran	15,508	60,312	10,318	49,994	5,190	0.1711	0.6653	0.2722
rnaSPAdes	15,508	177,248	9,441	167,807	6,067	0.0533	0.6088	0.0980
SOAPdenovo-Trans	15,508	158,690	9,324	149,366	6,184	0.0588	0.6012	0.1071
Trinity	15,508	61,208	9,444	42,660	6,064	**0.1813**	0.6088	**0.2794**
Merged[c]	15,508	375,854	11,178	364,676	4,330	0.0297	**0.7208**	0.0571
Test 3: Human HG38								
IDBA-Tran	17,669	52,368	9,301	43,067	8,368	0.1776	0.5264	0.2656
rnaSPAdes	17,669	249,621	10,216	239,405	7,453	0.0409	0.5782	0.0764
SOAPdenovo-Trans	17,669	124,507	9,318	115,189	8,351	0.0748	0.5274	0.1311
Trinity	17,669	40,728	9,603	31,125	8,066	**0.2358**	0.5435	**0.3289**
Merged[d]	17,669	392,627	11,267	381,360	6,402	0.0287	**0.6377**	0.0549

[a]The best performance score for each dataset is shown in red boldface.
[b]Total number of transcripts in the benchmark transcriptome.
[c]Number of unique contigs produced by the assembler.
[d]The Merged assembly is the union of all *de novo* assemblies across all kmer lengths

Table S6. Performance analysis of the three ensemble transcriptome assemblers.[a]

Assembler	Actual[b]	Total[c]	Unique[d]	*TP*	*FP*	*FN*	Precision	Recall	*F*
Tests 1: No0-NoAlt									
Concatenation	18,875	24,224	23,622 (125.15)	11,124	12,498	7,751	0.4709	0.5894	0.5235
EvidentialGene	18,875	85,845	78,765 (417.30)	12,519	66,246	6,356	0.1589	0.6633	0.2564
ConSemble3+d	18,875	20,263	20,263 (107.35)	13,352	6,911	5,523	**0.6589**	**0.7074**	**0.6823**
ConSemble2+d	18,875	51,117	51,117 (275.37)	13,979	37,998	4,896	0.2689	**0.7406**	0.3946
ConSemble4d	18,875	13,858	13,858 (73.42)	12,268	1,590	6,607	**0.8853**	0.6500	**0.7496**
Tests 2: Col0-Alt									
Concatenation	15,508	18,804	17,717 (114,24)	8,487	9,230	7,021	0.4790	0.5473	0.5109
EvidentialGene	15,508	46,841	43,821 (282.57)	7,218	36,603	8,290	0.1647	0.4654	0.2433
ConSemble3+d	15,508	17,309	17,309 (111.61)	9,189	8,120	6,319	**0.5309**	**0.5925**	**0.5600**
ConSemble2+d	15,508	44,243	44,243 (285.29)	10,261	33,982	5,247	0.2319	**0.6617**	0.3435
ConSemble4d	15,508	10,944	10,944 (70.57)	7,899	3,045	7,609	**0.7218**	0.5094	**0.5972**
Tests 3: Human HG38									
Concatenation	17,669	15,180	14,061 (79.58)	6,032	8,029	11,637	0.4290	0.3414	0.3802
EvidentialGene	17,669	65,587	59,221 (335.17)	6,248	52,973	11,421	0.1055	0.3536	0.1625
ConSemble3+d	17,669	19,349	19,349 (109.51)	9,128	10,221	8,541	**0.4718**	**0.5166**	**0.4932**
ConSemble2+d	17,669	42,961	42,961 (243.14)	10,191	32,770	7,478	0.2372	**0.5768**	0.3362
ConSemble4d	17,669	12,286	12,286 (69.53)	7,852	4,434	9,817	**0.6391**	0.4444	**0.5243**

[a]The best performance score for each dataset among Concatenation, EvidentialGene, and ConSemble3+d is shown in red boldface. When ConSemble2+d or ConSemble4d showed better performance than ConSemble3+d, such scores are shown in black boldface.

[b]Total number of transcripts in the benchmark transcriptome

[c]Number of all contigs produced by the assembler.

[d]Number of unique contigs produced by the assembler. Proportion (%) of the number of transcripts in the benchmark transcriptome is shown in parentheses.

Table S7. Performance analysis of genome-guided assemblers using the Tophat2 aligner.[a]

Assembler	Actual[b]	Total[c]	Unique[d]	*TP*	*FP*	*FN*	Precision	Recall	*F*
**[No0-NoAlt]**									
Test 4 (Reference: No-0)									
Bayesembler	18,875	15,297	15,171 (80.38)	11,200	3,971	7,675	0.7383	0.5934	0.6579
Cufflinks	18,875	19,398	19,288 (102.19)	14,531	4,757	4,344	**0.7534**	0.7699	**0.7615**
Scallop	18,875	21,853	21,397 (113.36)	15,184	6,213	3,691	0.7096	**0.8045**	0.7541
StringTie2	18,875	21,651	21,194 (112.29)	15,135	6,059	3,740	0.7141	0.8019	0.7554
Test 5 (Reference: Col-0)									
Bayesembler	18,875	15,670	15,531 (82.28)	5,142	10,389	13,733	**0.3311**	0.2724	0.2989
Cufflinks	18,875	20,133	19,938 (105.63)	6,510	13,428	12,365	0.3265	0.3449	0.3355
Scallop	18,875	22,804	22,298 (118.14)	6,908	15,390	11,967	0.3098	0.3660	0.3356
StringTie2	18,875	22,275	21,767 (115.32)	6,857	14,910	12,018	0.3150	**0.3633**	**0.3374**
**[Col0-Alt]**									
Test 6 (Reference: Col-0)									
Bayesembler	15,508	16,271	15,143 (97.65)	9,158	5,985	6,350	**0.6048**	0.5905	0.5976
Cufflinks	15,508	16,232	15,768 (101.68)	8,560	7,208	6,948	0.5429	0.5520	0.5474
Scallop	15,508	19,454	18,055 (116.42)	10,534	7,521	4,974	0.5834	**0.6793**	**0.6277**
StringTie2	15,508	18,897	17,721 (114.27)	10,034	7,687	5,474	0.5662	0.6470	0.6039
Test 7 (Reference: No-0)									
Bayesembler	15,508	16,384	15,329 (98.85)	4,810	10,519	10,698	**0.3138**	0.3102	0.3120
Cufflinks	15,508	17,303	16,662 (107.44)	4,321	12,341	11,187	0.2593	0.2786	0.2686
Scallop	15,508	19,539	18,408 (118.70)	5,315	13,093	10,193	0.2887	**0.3427**	**0.3134**
StringTie2	15,508	19,505	18,332 (118.21)	5,199	13,133	10,309	0.2836	0.3252	0.3073
**[Human HG38]**									
Test 8 (Reference: HG38)									
Bayesembler	17,669	15,424	13,919 (78.78)	7,524	6,395	10,145	**0.5406**	0.4258	0.4764
Cufflinks	17,669	15,135	14,923 (84.46)	7,280	7,643	10,389	0.4878	0.4120	0.4467
Scallop	17,669	29,325	26,857 (152.00)	8,642	18,215	9,027	0.3218	0.4891	0.3882
StringTie2	17,669	23,623	19,353 (109.53)	8,976	10,377	8,693	0.4638	**0.5080**	**0.4849**
Test 9 (Reference: HX1)									
Bayesembler	17,669	15,424	14,610 (82.69)	5,413	9,197	12,256	**0.3705**	0.3064	0.3354
Cufflinks	17,669	16,554	16,258 (92.01)	5,296	10,962	12,373	0.3257	0.2997	0.3122
Scallop	17,669	19,980	18,779 (106.28)	6,132	12,646	11,536	0.3266	0.3471	**0.3365**
StringTie2	17,669	21550	20,202 (114.34)	6,217	13,985	11,452	0.3077	**0.3519**	0.3283

[a]The best performance score for each dataset is shown in red boldface.
[b]Total number of transcripts in the benchmark transcriptome.
[c]Number of all contigs produced by the assembler.
[d]Number of unique contigs produced by the assembler. Proportion (%) of the number of transcripts in the benchmark transcriptome is shown in parentheses.

Table S8. Performance analysis of genome-guided assemblers using the STAR aligner.

Assembler	Actual[a]	Total[b]	Unique[c]	*TP*	*FP*	*FN*	Precision	Recall	*F*
**[No0-NoAlt]**									
Test 4 (Reference: No-0)									
Bayesembler	18,875	-	-	-	-	-	-	-	-
Cufflinks	18,875	21,327	20,925 (110.86)	13,769	7,156	5,106	0.6580	0.7295	0.6919
Scallop	18,875	19,714	19,660 (104.16)	15,652	4,008	3,223	0.7961	0.8292	0.8124
StringTie2	18,875	19,920	19,668 (104.20)	15,930	3,738	2,945	**0.8099**	**0.8440**	**0.8266**
Test 5 (Reference: Col-0)									
Bayesembler	18,875	-	-	-	-	-	-	-	-
Cufflinks	18,875	21,722	21,178 (112.20)	5,953	15,225	12,922	0.2811	0.3154	0.2973
Scallop	18,875	20,385	20,264 (107.36)	7,092	13,172	11,783	0.3500	**0.3757**	0.3624
StringTie2	18,875	18,150	17,212 (91.19)	6,625	10,587	12,250	**0.3849**	0.3510	**0.3672**
**[Col0-Alt]**									
Test 6 (Reference: Col-0)									
Bayesembler	15,508	-	-	-	-	-	-	-	-
Cufflinks	15,508	18,060	17,414 (112.29)	7,959	9,455	7,549	0.4570	0.5132	0.4835
Scallop	15,508	18,188	17,179 (110.78)	10,468	6,711	5,040	**0.6093**	**0.6750**	**0.6405**
StringTie2	15,508	18,018	17,000 (109.62)	10,061	6,939	5,447	0.5918	0.488	0.6190
Test 7 (Reference: No-0)									
Bayesembler	15,508	-	-	-	-	-	-	-	-
Cufflinks	15,508	18,218	17,441 (112.46)	3,977	13,464	11,531	0.2280	0.2564	0.2414
Scallop	15,508	18,176	17,219 (111.03)	5,293	11,926	10,215	**0.3074**	**0.3413**	**0.3235**
StringTie2	15,508	18,515	17,477 (112.70)	5,169	12,308	10,339	0.2958	0.3333	0.3134
**[Human HG38]**									
Test 8 (Reference: HG38)									
Bayesembler	17,669	-	-	-	-	-	-	-	-
Cufflinks	17,669	15,206	14,495 (82.04)	7,628	6,867	10,041	0.5263	0.4317	0.4743
Scallop	17,669	17,940	17,054 (96.52)	9,309	7,745	8,360	**0.5459**	0.5269	0.5362
StringTie2	17,669	22,546	18,237 (103.21)	9,761	8,476	7,908	0.5352	**0.5524**	**0.5437**
Test 9 (Reference: HX1)									
Bayesembler	17,669	-	-	-	-	-	-	-	-
Cufflinks	17,669	17,405	16,438 (93.03)	5,491	10,947	12178	0.3340	0.3108	0.3220
Scallop	17,669	19,499	18,477 (104.57)	6,557	11,920	11112	**0.3549**	0.3711	0.3628
StringTie2	17,669	20,587	19,309 (109.28)	6,757	12,552	10,912	0.3499	**0.3824**	**0.3655**

[a]The best performance score for each dataset is shown in red boldface.
[b]Total number of transcripts in the benchmark transcriptome.
[c]Number of all contigs produced by the assembler.
[d]Number of unique contigs produced by the assembler. Proportion (%) of the number of transcripts in the benchmark transcriptome is shown in parentheses.
[e]Bayesembler requires Tophat2 alignments, and as of this writing cannot run on alignments produced by STAR.

Table S9. Performance analysis of the three ensemble transcriptome assemblers.[a]

Assembler	Actual[b]	Total[c]	Unique[d]	*TP*	*FP*	*FN*	Precision	Recall	*F*
Test 4: No0-Alt (Reference: No0)									
TransBorrow	18,875	23,982	22,592 (119.69)	15,689	6,903	3,186	0.6944	0.8312	0.7567
ConSemble3+g	18,875		15,688 (83.12)	14,200	1,488	4,675	**0.9052**	**0.7523**	**0.8217**
ConSemble2+g	18,875		19,947 (105.68)	15,819	4,128	3,056	0.7931	**0.8381**	0.8150
ConSemble4g	18,875		9,922 (52.57)	9,416	506	9,459	**0.9490**	0.4989	0.6540
Test 6: Col0-Alt (Reference: Col0)									
TransBorrow	15,508	1,819	1,800 (11.61)	1,019	781	14,489	0.5661	0.0657	0.1177
ConSemble3+g	15,508	13,380	13,380 (86.28)	9,679	3,701	5,829	**0.7234**	**0.6241**	**0.6701**
ConSemble2+g	15,508	17,200	17,200 (110.91)	10,754	6,446	4,754	0.6252	**0.6934**	0.6576
ConSemble4g	15,508	7,968	7,968 (51.38)	6,328	1,640	9,180	**0.7942**	0.4080	0.5391
Test 8: Human HG38 (Reference: HG38)									
TransBorrow	17,669	21,422	18,605 (105.30)	8,823	9,782	8,846	0.4742	**0.4993**	0.4865
ConSemble3+g	17,669	11,945	11,945 (67.60)	7,744	4,201	9,925	**0.6483**	0.4383	**0.5230**
ConSemble2+g	17,669	16,127	16,127 (91.27)	8,864	7,263	8,805	0.5496	**0.5017**	**0.5246**
ConSemble4g	17,669	7,713	7,713 (43.65)	5,670	2,043	11,999	**0.7351**	0.3209	0.4468

[a]The better performance score for each dataset between TransBorrow and ConSemble3+g is shown in red boldface. When ConSemble2+g or ConSemble4g showed better performance than ConSemble3+g, such scores are shown in black boldface.

[b]Total number of transcripts in the benchmark transcriptome

[c]Number of all contigs produced by the assembler.

[d]Number of unique contigs produced by the assembler. Proportion (%) of the number of transcripts in the benchmark transcriptome is shown in parentheses.

Table S10. Performance analysis of transcriptome assembly at the nucleotide level.[a]

Assembler	No0-NoAlt					Col0-Alt					Human HG38				
	F[b]	RSEM-EVAL ($x10^{-9}$)	Nucleotide F_1	Contig F_1	KC	F[b]	RSEM-EVAL ($x10^{-9}$)	Nucleotide F_1	Contig F_1	KC	F[b]	RSEM-EVAL ($x10^{-9}$)	Nucleotide F_1	Contig F_1	KC
**[*De novo*]**															
IDBA-Tran	0.40	-3.91	**0.95**	0.26	0.89	0.33	-3.98	0.82	0.19	0.82	0.32	-3.50	**0.77**	0.14	0.67
rnaSPAdes	0.43	-2.44	0.71	0.42	0.87	0.32	-2.31	0.64	0.19	0.87	0.39	-2.32	0.72	0.26	0.68
SOAPdenovo-Trans	0.46	-2.31	0.92	**0.49**	0.86	0.39	-2.28	0.82	0.36	**0.88**	0.30	-2.24	**0.77**	0.25	0.68
Trinity	**0.57**	**-2.30**	0.93	0.48	**0.90**	**0.53**	**-1.88**	**0.85**	**0.52**	0.86	**0.45**	**-1.76**	0.75	**0.35**	**0.70**
**[Genome-guided using the same reference]**															
Bayesembler	0.66	-3.34	0.78	0.58	0.71	0.60	-2.86	0.76	0.59	0.69	**0.48**	-2.56	0.72	**0.40**	0.72
Cufflinks	**0.76**	-1.98	**0.95**	0.57	0.90	0.54	-2.00	0.83	0.51	**0.89**	0.45	-2.72	0.81	0.34	0.76
Scallop	0.75	-1.69	0.88	**0.74**	**0.92**	**0.63**	-1.62	**0.86**	**0.67**	0.86	0.39	-1.99	0.73	0.35	0.85
StringTie2	0.75	**-0.97**	0.90	0.63	0.91	0.60	**-0.96**	0.83	0.61	0.84	**0.48**	**-1.05**	**0.76**	0.38	**0.86**
**[Ensemble]**															
Concatenation	0.52	-1.93	0.83	0.53	0.86	0.51	-1.98	0.79	**0.51**	0.81	0.38	-2.62	0.68	0.30	0.57
EvidentialGene	0.26	-2.08	0.36	0.18	0.71	0.24	-2.56	0.50	0.17	0.72	0.16	-2.49	0.47	0.07	0.57
TransBorrow	0.76	**-0.88**	**0.84**	**0.58**	**0.91**	0.12	-4.77	0.18	0.15	0.11	0.49	**-1.03**	**0.73**	**0.38**	**0.85**
ConSemble3+d	0.68	-3.29	0.82	0.21	0.80	0.56	-3.78	0.78	0.14	0.73	0.49	-3.59	0.68	0.08	0.59
ConSemble3+g	**0.82**	-1.88	0.70	0.51	0.65	**0.67**	**-1.67**	0.73	0.50	0.62	**0.52**	-1.93	0.68	0.33	0.60
ConSemble3+dLong[c]	0.68	-2.18	**0.84**	0.54	0.86	0.56	-1.96	0.81	0.48	**0.83**	0.49	-1.81	0.66	0.32	0.67
ConSemble3+dHigh[d]	0.68	-2.27	**0.84**	0.51	0.85	0.56	-2.05	**0.82**	0.45	0.82	-	-	-	-	-

[a]The best score among the assemblers for each group is shown in red boldface.
[b]From Table S3 and Tables S6 (for *de novo* assemblers) and Tests 4, 6, and 8 in Tables S7 and S9 (for genome-guided assemblers).
[c]Longest contigs producing the protein sequences kept by ConSemble are chosen.
[d]Contigs with the highest RSEM-EVAL producing the protein sequences kept by ConSemble are chosen.

Table S11. Performance analysis for the No0-NoAlt assembly using different identity thresholds.[a]

Assembler	*TP*			Precision			Recall			*F*		
	95%	98%	100%	95%	98%	100%	95%	98%	100%	95%	98%	100%
**[Genome-guided using the same reference]**												
Bayesembler	11,493	11,390	11,200	0.7575	0.7507	0.7382	0.6089	0.6034	0.5934	0.6751	0.6691	0.6579
Cufflinks	15,134	14,871	14,531	**0.7846**	**0.7710**	**0.7534**	0.8018	0.7879	0.7699	0.7931	0.7793	**0.7615**
Scallop	15,537	15,433	**15,184**	0.7261	0.7213	0.7096	0.8232	0.8176	**0.8045**	0.7716	0.7664	0.7541
StringTie	**16,304**	**15,812**	15,135	0.7530	0.7303	0.6990	**0.8638**	**0.8377**	0.8019	**0.8046**	**0.7803**	0.7469
**[Genome-guided using the different reference]**												
Bayesembler	10,191	9,780	5,142	0.6562	0.6297	**0.3311**	0.5399	0.5181	0.2724	0.5924	0.5685	0.2989
Cufflinks	13,683	12,779	6,510	**0.6863**	**0.6409**	0.3265	0.7249	0.6770	0.3449	0.7051	**0.6585**	0.3355
Scallop	13,537	13,003	**6,908**	0.6071	0.5831	0.3098	0.7172	0.6889	**0.3660**	0.6576	0.6316	**0.3356**
Stringtie	**14,632**	**13,548**	6,857	0.6569	0.6082	0.3078	**0.7752**	**0.7178**	0.3633	**0.7112**	**0.6585**	0.3333
**[*De novo*]**												
IDBA-Tran	10,368	9,120	8,344	0.4545	0.3998	0.3658	0.5493	0.4832	0.4421	0.4974	0.4375	0.4003
rnaSPAdes	13,505	12,452	10,034	0.4873	0.4493	0.3621	**0.7155**	0.6597	0.5316	0.5798	0.5345	0.4307
SOAPdenovo	11,931	11,643	11,118	0.3994	0.3897	0.3721	0.6321	0.6168	0.5890	0.4895	0.4777	0.4561
Trinity	**12,950**	**12,588**	**12,057**	**0.5506**	**0.5352**	**0.5126**	0.6861	**0.6669**	**0.6388**	**0.6109**	**0.5939**	**0.5688**
**[Ensemble]**												
Concatenation	13,973	13,662	11,124	0.5915	0.5784	0.4709	0.7403	0.7238	0.5894	0.6576	0.6430	0.5235
EvidentialGene	15,216	14,493	12,519	0.1932	0.1840	0.1589	0.8061	0.7678	0.6633	0.3117	0.2969	0.2564
ConSemble3+d	13,695	13,600	13,352	0.6747	0.6700	0.6578	0.7256	0.7205	0.7074	**0.6992**	**0.6944**	**0.6823**
ConSemble3+g	14,630	14,519	14,223	**0.9326**	**0.9255**	**0.9066**	0.7751	0.7692	0.7535	**0.8466**	**0.8401**	**0.8230**
TransBorrow	**17,154**	**16,539**	**15,689**	0.7153	0.6896	0.6542	**0.9088**	**0.8762**	**0.8312**	0.8005	0.7718	0.7322

[a]The best score among the assemblers for each group is shown in red boldface. For *de novo* ensemble methods, the best *F* scores are shown in blue boldface.

Table S12. Performance analysis for the Col0-Alt assembly using different identity thresholds.[a]

Assembler	*TP*			Precision			Recall			*F*		
	95%	98%	100%	95%	98%	100%	95%	98%	100%	95%	98%	100%
**[Genome-guided using the same reference]**												
Bayesembler	10,403	9,906	9,158	**0.6869**	**0.6541**	**0.6047**	0.6708	0.6388	0.5905	0.6788	0.6464	0.5975
Cufflinks	10,312	9,525	8,560	0.6539	0.6040	0.5428	0.6649	0.6142	0.5520	0.6594	0.6091	0.5474
Scallop	12,281	**11,615**	**10,534**	0.6802	0.6433	0.5834	0.7919	**0.7490**	**0.6793**	**0.7318**	**0.6921**	**0.6277**
StringTie	**12,284**	11,370	10,034	0.6501	0.6017	0.5310	**0.7921**	0.7332	0.6470	0.7141	0.6610	0.5833
**[Genome-guided using the different reference]**												
Bayesembler	9,083	8,422	4,810	**0.5925**	**0.5494**	**0.3138**	0.5857	0.5431	0.3102	0.5891	0.5462	0.3120
Cufflinks	8,846	7,811	4,321	0.5309	0.4688	0.2593	0.5704	0.5037	0.2786	0.5499	0.4856	0.2686
Scallop	10,224	9,366	**5,315**	0.5554	0.5088	0.2887	0.6593	0.6039	**0.3427**	0.6029	**0.5523**	**0.3134**
Stringtie	**10,692**	**9,530**	5,199	0.5482	0.4886	0.2665	**0.6895**	**0.6145**	0.3352	**0.6107**	0.5444	0.2970
**[*De novo*]**												
IDBA-Tran	7,634	6,842	6,021	0.3733	0.3346	0.2945	0.4923	0.4412	0.3883	0.4246	0.3806	0.3349
rnaSPAdes	9,677	8,827	7,556	0.3073	0.2803	0.2399	0.6240	0.5692	0.4872	0.4118	0.3756	0.3215
SOAPdenovo	8,940	8,222	7,281	0.4183	0.3847	0.3407	0.5765	0.5302	0.4695	0.4848	0.4459	0.3948
Trinity	**11,147**	**10,457**	**9,252**	**0.5743**	**0.5387**	**0.4767**	**0.7188**	**0.6743**	**0.5966**	**0.6385**	**0.5989**	**0.5299**
**[Ensemble]**												
Concatenation	10,463	9,744	8,487	0.5906	0.5500	0.4790	0.6747	0.6283	0.5473	0.6298	0.5865	0.5109
EvidentialGene	10,083	8,967	7,218	0.2301	0.2046	0.1647	0.6502	0.5782	0.4654	0.3399	0.3023	0.2433
ConSemble3+d	10,777	10,184	9,189	0.6214	0.5872	0.5299	0.6949	0.6567	0.5927	**0.6561**	**0.6200**	**0.5600**
ConSemble3+g	**11,337**	**10,752**	**9,793**	**0.8473**	**0.8036**	**0.7319**	**0.7310**	**0.6933**	**0.6315**	**0.7849**	**0.7444**	**0.6780**
TransBorrow	1,358	1,228	1,019	0.7466	0.6751	0.5602	0.0876	0.0792	0.0657	0.1567	0.1417	0.1176

[a]The best score among the assemblers for each group is shown in red boldface. For *de novo* ensemble methods, the best *F* scores are shown in blue boldface.

Table S13. Performance analysis for the Human HG38 assembly using different identity thresholds.[a]

Assembler	*TP*			Precision			Recall			*F*		
	95%	98%	100%	95%	98%	100%	95%	98%	100%	95%	98%	100%
**[Genome-guided using the same reference]**												
Bayesembler	8,881	8,533	7,524	**0.6380**	**0.6130**	**0.5405**	0.5026	0.4829	0.4258	**0.5623**	**0.5403**	**0.4764**
Cufflinks	8,690	8,209	7,280	0.5823	0.5501	0.4878	0.4918	0.4646	0.4120	0.5333	0.5037	0.4467
Scallop	10,175	9,761	8,642	0.3788	0.3634	0.3218	0.5759	0.5524	0.4891	0.4570	0.4384	0.3882
StringTie	**11,166**	**10,460**	**8,976**	0.4727	0.4428	0.3800	**0.6320**	**0.5920**	**0.5080**	0.5408	0.5066	0.4348
**[Genome-guided using the different reference]**												
Bayesembler	7,486	7,061	5,413	**0.5124**	**0.4833**	**0.3705**	0.4237	0.3996	0.3064	**0.4638**	**0.4375**	0.3354
Cufflinks	7,399	6,855	5,296	0.4551	0.4216	0.3257	0.4188	0.3880	0.2997	0.4362	0.4041	0.3122
Scallop	8,406	7,938	6,132	0.4476	0.4227	0.3265	0.4757	0.4493	0.3470	0.4613	0.4356	**0.3365**
Stringtie	**8,888**	**8,199**	**6,217**	0.4124	0.3805	0.2885	**0.5030**	**0.4640**	**0.3519**	0.4532	0.4181	0.3170
**[*De novo*]**												
IDBA-Tran	7,750	7,177	6,154	0.3698	0.3425	0.2937	0.4386	0.4062	0.3483	0.4013	0.3716	0.3187
rnaSPAdes	9,727	9,034	7,637	0.4579	0.4252	0.3595	0.5505	0.5113	0.4322	0.4999	0.4643	0.3925
SOAPdenovo	7,614	7,071	5,933	0.3460	0.3213	0.2696	0.4309	0.4002	0.3358	0.3838	0.3564	0.2991
Trinity	**10,385**	**9,980**	**8,764**	**0.4881**	**0.4690**	**0.4119**	**0.5878**	**0.5648**	**0.4960**	**0.5333**	**0.5125**	**0.4500**
**[Ensemble]**												
Concatenation	7,497	7,078	6,032	0.5332	0.5034	0.4290	0.4243	0.4006	0.3414	0.4725	0.4461	0.3802
EvidentialGene	8,775	7,895	6,248	0.1482	0.1333	0.1055	0.4966	0.4468	0.3536	0.2282	0.2054	0.1625
ConSemble3+d	10,673	**10,315**	**9,128**	0.5516	0.5331	0.4717	0.6041	**0.5838**	**0.5166**	**0.5766**	**0.5573**	**0.4932**
ConSemble3+g	9,447	9,066	8,045	**0.7909**	**0.7590**	**0.6735**	0.5347	0.5131	0.4553	**0.6380**	**0.6123**	**0.5433**
TransBorrow	**10,982**	10,273	8,823	0.5127	0.4796	0.4119	**0.6215**	0.5814	0.4993	0.5619	0.5256	0.4514

[a]The best score among the assemblers for each group is shown in red boldface. For *de novo* ensemble methods, the best *F* scores are shown in blue boldface.
