# Supplementary material for: A consensus-based ensemble approach to improve transcriptome assembly
Source: BMC Bioinformatics. 2021 Oct 21;22:513. doi: 10.1186/s12859-021-04434-8 (PMC8532302; doi:10.1186/s12859-021-04434-8)
Supplement: Supplementary file 3 — Additional file 3. Supplementary Figures (S1–S4). [file 12859_2021_4434_MOESM3_ESM.pdf]

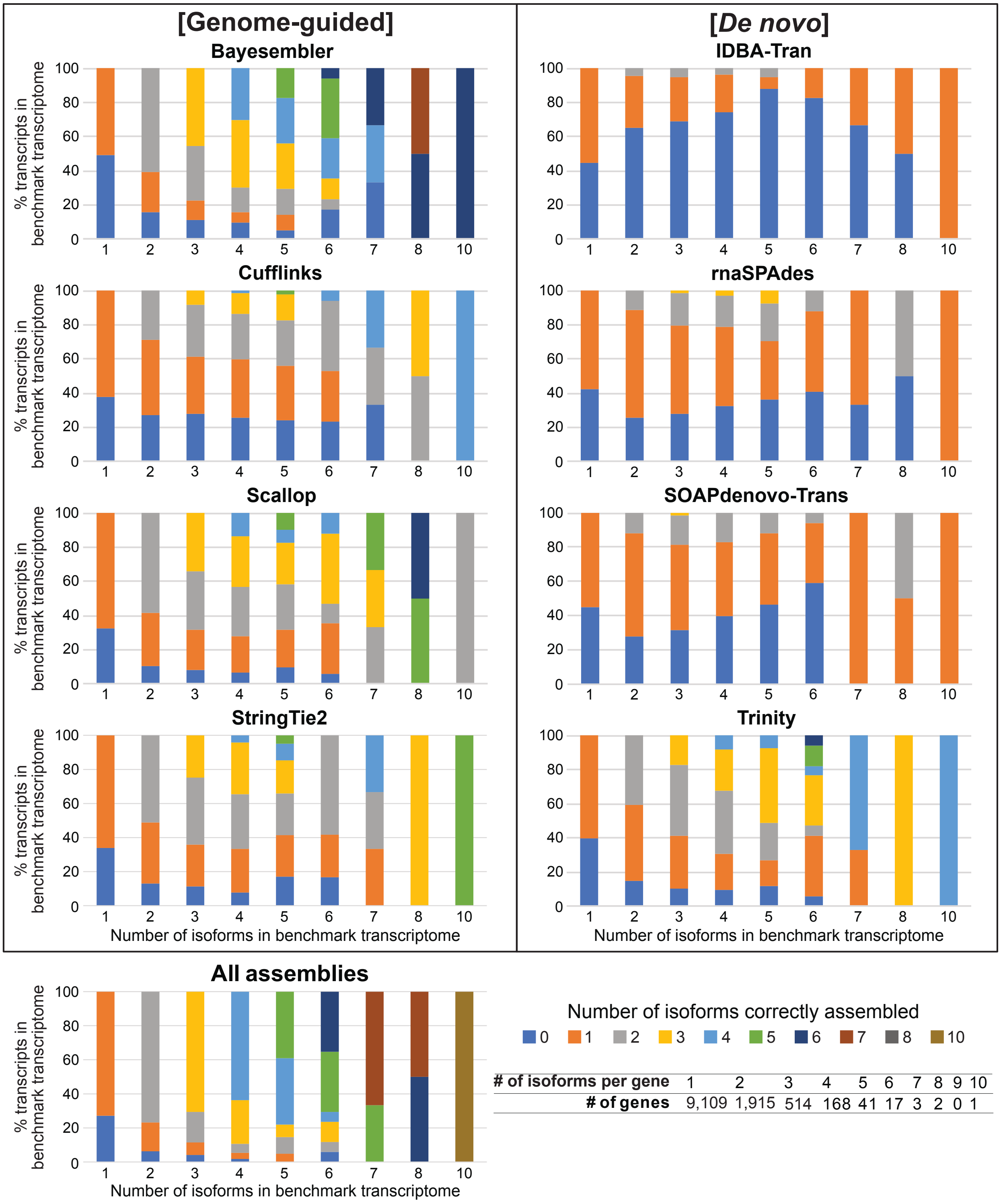

Fig. S1. Proportion (%) of correctly assembled isoforms per gene by different assemblers. Genes are grouped by the number of the isoforms existing in the reference. For each group, the proportion for each assembled isoform number (from 0 to 9) is color coded where 0 indicates no transcripts were assembled for a given gene. Results are based on Test 3 (Col0-Alt using the Col-0 reference) for genome-guided and Test 8 (Col0-Alt) for *de novo* assembly (with default settings) in Table S2 in Additional file 2. In "All assemblies", all genome-guided and *de novo* assemblies across all kmer lengths were merged.

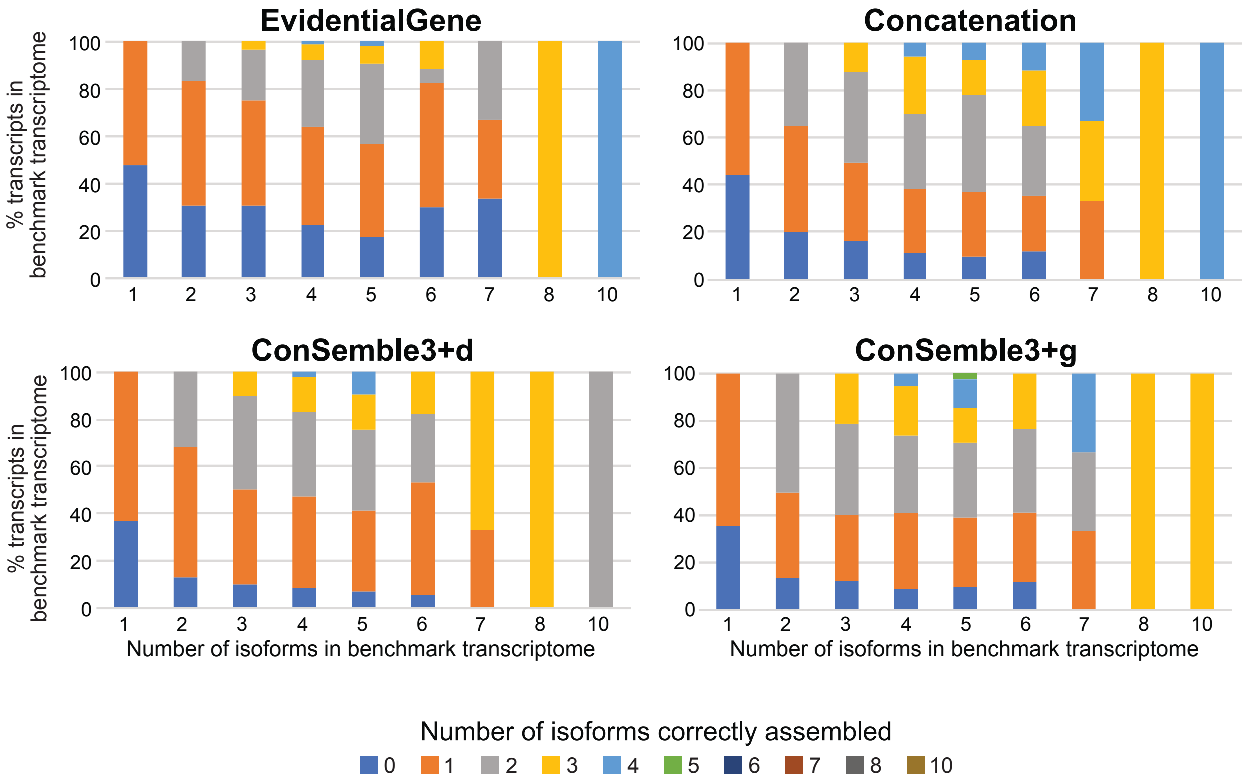

# of isoforms per gene	1	2	3	4	5	6	7	8	9	10
# of genes	9,109	1,915	514	168	41	17	3	2	0	1

Fig. S2. Proportion of correctly assembled isoforms per gene by different ensemble assemblers. Genes are grouped by the number of isoforms existing in the reference. For each group, the proportion for each assembled isoform number (from 0 to 9) is color coded where 0 indicates no transcripts were assembled for a given gene. Results are based on Test 3 (Col0-Alt using Col-0 reference) for ConSemble3+g and Test 8 (Col0-Alt) for EvidentialGene, Concatenation, and ConSemble3+d (see Table S2 in Additional file 2).

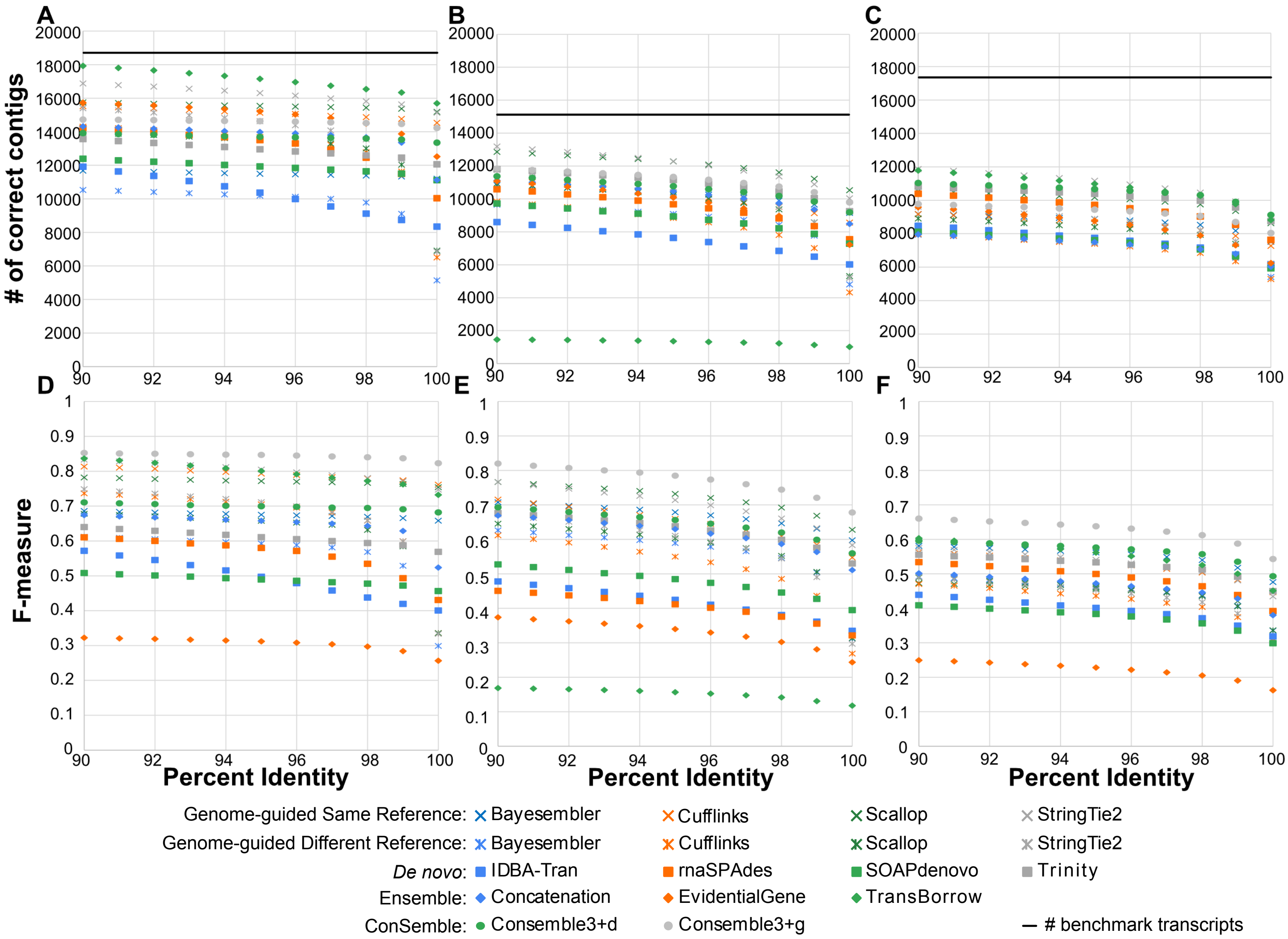

Fig. S3. The numbers of correctly assembled contigs with varied percent identity threshold for true positives. The threshold % identity was varied from 90% to 100% (at the protein level). The number of correctly assembled contigs was counted for each % identity threshold (A, B, and C). These correctly assembled contigs for each threshold were also used to calculate the F-measure (D, E, and F). The dataset used was No0-NoAlt (A and D), Col0-Alt (B and E), or Human HG38 (C and F). See Tables S11-S13 in Additional file 2 for more statistics.

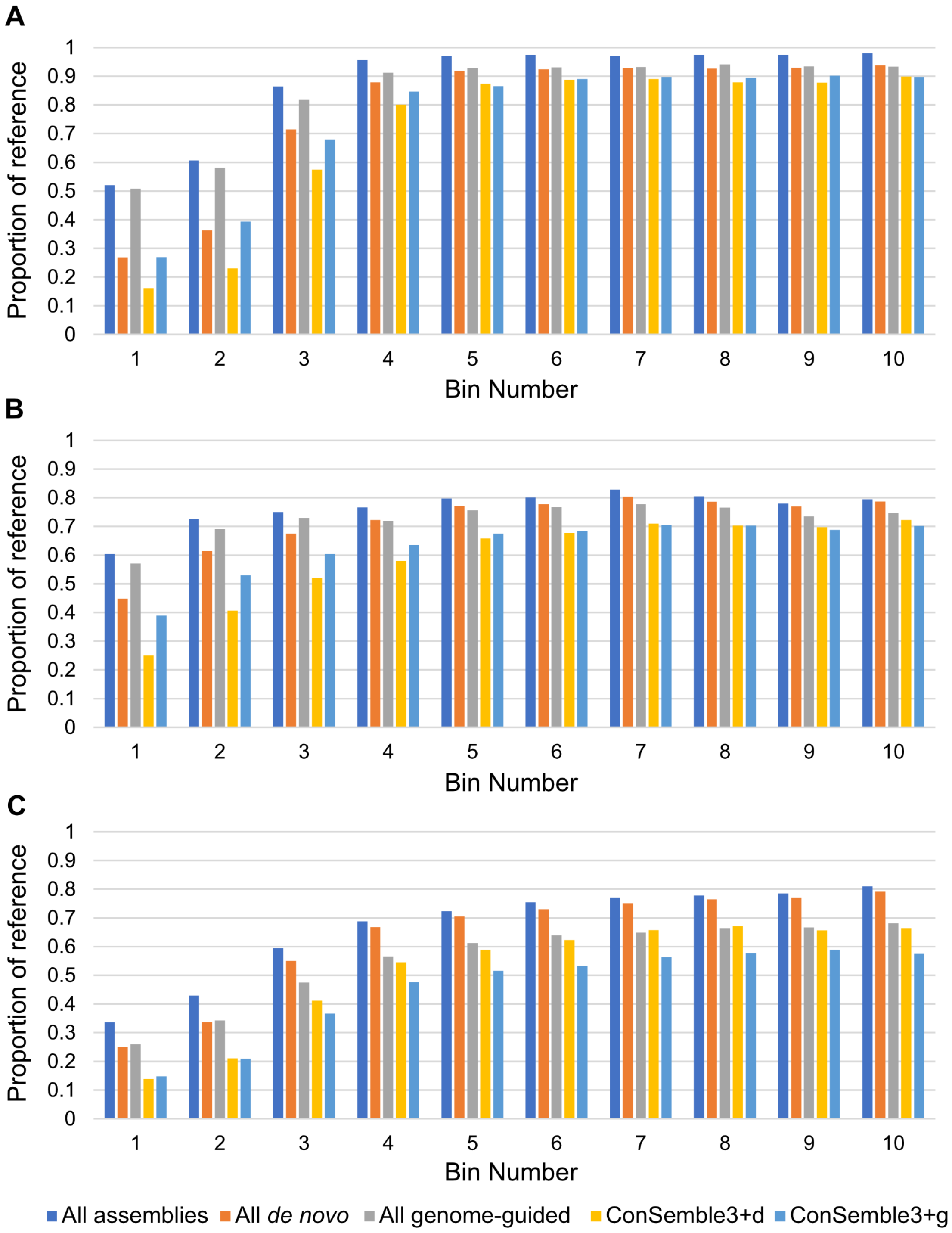

Fig. S4. Proportion of correctly assembled transcripts by expression. Genes are grouped into ten equally sized bins by expression decile in the benchmark datasets (A: No0-NoAlt, B: Col0-Alt, and C: Human HG38). For each bin, the proportion of benchmark transcripts correctly assembled for each of the following categories are shown: "All assemblies": transcript is correctly assembled by any *de novo* or genome-guided methods; "All *de novo*": transcript is correctly assembled by any *de novo* method, regardless of the kmer length; "All genome-guided": transcript is correctly assembled by any genome-guided method; "ConSemble3+d": transcript is correctly assembled by ConSemble3+d; and "ConSemble3+g": transcript is correctly assembled by ConSemble3+g.
